# Reduced Expression of Oligodendrocyte Linage-Enriched Transcripts During the Endoplasmic Reticulum Stress/Integrated Stress Response

**DOI:** 10.1080/17590914.2024.2371162

**Published:** 2024-07-16

**Authors:** Yonglin Gao, Lukasz P. Slomnicki, Ewa Kilanczyk, Michael D. Forston, Maciej Pietrzak, Eric C. Rouchka, Russell M. Howard, Scott R. Whittemore, Michal Hetman

**Affiliations:** a Kentucky Spinal Cord Injury Research Center, University of Louisville School of Medicine, Louisville, Kentucky, USA; b Departments of Neurological Surgery, University of Louisville School of Medicine, Louisville, Kentucky, USA; c Department of Biomedical Informatics, The Ohio State University, Columbus, Ohio, USA; d Kentucky Biomedical Research Infrastructure Network Bioinformatics Core, University of Louisville, Louisville, Kentucky, USA; e Anatomical Sciences & Neurobiology, University of Louisville School of Medicine, Louisville, Kentucky, USA; f Pharmacology & Toxicology, University of Louisville School of Medicine, Louisville, Kentucky, USA; g Biochemistry and Molecular Genetics, University of Louisville School of Medicine, Louisville, Kentucky, USA

**Keywords:** Differentiation, endoplasmic reticulum stress, integrated stress response, myelination, oligodendrocyte precursor cells, transcriptome, white matter injury

## Abstract

Endoplasmic reticulum (ER) stress in oligodendrocyte (OL) linage cells contributes to several CNS pathologies including traumatic spinal cord injury (SCI) and multiple sclerosis. Therefore, primary rat OL precursor cell (OPC) transcriptomes were analyzed using RNASeq after treatments with two ER stress-inducing drugs, thapsigargin (TG) or tunicamycin (TM). Gene ontology term (GO) enrichment showed that both drugs upregulated mRNAs associated with the general stress response. The GOs related to ER stress were only enriched for TM-upregulated mRNAs, suggesting greater ER stress selectivity of TM. Both TG and TM downregulated cell cycle/cell proliferation-associated transcripts, indicating the anti-proliferative effects of ER stress. Interestingly, many OL lineage-enriched mRNAs were downregulated, including those for transcription factors that drive OL identity such as *Olig2*. Moreover, ER stress-associated decreases of OL-specific gene expression were found in mature OLs from mouse models of white matter pathologies including contusive SCI, toxin-induced demyelination, and Alzheimer’s disease-like neurodegeneration. Taken together, the disrupted transcriptomic fingerprint of OL lineage cells may facilitate myelin degeneration and/or dysfunction when pathological ER stress persists in OL lineage cells.

## SUMMARY STATEMENT

The ER stress response compromises the transcriptomic identity of the OL lineage. Therefore, persistent, pathological ER stress may have a negative impact on structural and/or functional integrity of the white matter.

## Introduction

Endoplasmic reticulum (ER) stress follows disturbed ER function that results in the accumulation of unfolded proteins in the ER (Hetz & Papa, 2018; Walter & Ron, 2011). As secreted and/or membrane proteins pass through the ER during their maturation, cell types with high levels of membrane synthesis/protein secretion, including oligodendrocytes (OLs), are prone to ER stress (Lin & Popko, 2009). Although the ER stress response (ERSR) initiates several adaptive mechanisms to restore ER function, its prolonged activation may be cytotoxic (Hetz & Papa, 2018). Both the adaptive- and the cytotoxic arm of the ERSR involve complex changes in gene expression that are mediated by translational regulation, mRNA processing/degradation, and transcription factor (TF) activation. Phosphorylation of eukaryotic initiation factor 2α (eIF2α), which also occurs in response to other, non-ER stressors, transiently inhibits general protein synthesis, reducing nascent protein influx into the ER while increasing translation of the integrated stress response (ISR) TFs ATF4 and CHOP/DDIT3. Expression of the latter TF is further enhanced by ATF4-mediated transcription (Hetz & Papa, 2018; Pakos-Zebrucka et al., 2016). Activation of the nuclease IRE1 triggers the splicing of mRNA for the unfolded protein response (UPR) TF XBP1(Maurel et al., 2014). If IRE1 activation persists, it degrades transcripts of several pro-survival genes triggering apoptosis (Maurel et al., 2014). Other TFs including ATF6 and NFκB are also activated (Hetz & Papa, 2018; Lei et al., 2020; Pakos-Zebrucka et al., 2016). While the adaptive or cytotoxic consequences of their activity may be cell type-dependent, the ISR TFs (ATF4, CHOP) are often implicated in ER stress toxicity and the UPR TFs (XBP1, ATF6) are usually adaptive (Hetz & Papa, 2018; Maurel et al., 2014; Ron & Walter, 2007). The effector mechanisms of the cytotoxic ERSR/ISR may involve the induction of specific pro-apoptotic genes or an anabolic gene expression program including amino acyl tRNA synthetases, translation factors, and amino acid transporters to promote recovery of ER protein synthesis (Han et al., 2013; Hetz & Papa, 2018; Krokowski et al., 2013). Without timely restoration of ER function, such a program causes irreversible ER damage resulting in cell death (Han et al., 2013; Krokowski et al., 2013). Target effector genes of the adaptive TFs include ER chaperones and many components of the ER-associated protein degradation pathway (ERAD) (Hetz & Papa, 2018; Walter & Ron, 2011).

OL ER stress is documented in various types of white matter pathologies including genetic degenerative leukodystrophies, neuroinflammation-associated damage in multiple sclerosis (MS) or demyelination after contusive spinal cord injury (SCI) (Lin & Popko, 2009; Whittemore et al., 2022). Enhancement of the adaptive ERSR and/or blocking the cytotoxic ERSR protects white matter in those conditions (Lin et al., 2013; Ohri et al., 2011, 2013; Lin et al., 2014). Conversely, white matter damage is enhanced when the ERSR pro-homeostatic arm is blocked (Hussien et al., 2014; Lei et al., 2020; Saraswat Ohri et al., 2021; Stone et al., 2017). Contributions of OL transcriptome changes to the outcome of ER stress are exemplified by deleterious consequences of OL-specific inhibition of the ERSR TFs sXBP1 or NFκB in SCI or experimental autoimmune encephalitis (EAE), respectively (Stone et al., 2017; Saraswat Ohri et al., 2021). However, large-scale analyses of OL transcriptome under ER stress have not been reported.

Tunicamycin (TM) and thapsigargin (TG) are two cytotoxic drugs that are widely used to induce ER stress (Foufelle & Fromenty, 2016). However, their respective ER stress induction mechanisms are different. TM inhibits dolichyl-phosphate N-acetylglucosaminephosphotransferase 1 (DPAGT1) disrupting the first step in N-linked protein glycosylation and perturbing ER folding of glycoproteins (Foufelle & Fromenty, 2016). However, besides inducing ER protein misfolding, it can also reduce expression/activity of such critical glycoproteins as the growth/neurotrophic factor receptor tyrosine kinases (Watson et al., 1999). TG blocks the sarcoplasmic/endoplasmic reticulum Ca^2+^-ATPase (SERCA) reducing ER [Ca^2+^] and inducing ER protein misfolding by perturbing activity of Ca^2+^-dependent chaperones (Foufelle & Fromenty, 2016). While the TG-induced depletion of ER Ca^2+^ triggers ER protein misfolding, accumulation of Ca^2+^ in the cytosol activates Ca^2+^ signaling (Pick et al., 2023; Razani-Boroujerdi et al., 1994; Rodland et al., 1997). Therefore, neither TM nor TG are selective ER stress inducers.

This study was initiated to analyze transcriptomic effects of ER stress in primary rat OL precursor cells (OPCs) which are a widely used cell culture model of the OL linage. As TM and TG have both ER stress- and non-ER stress dependent effects, transcriptomic changes in response to each of these drugs were determined and compared.

## Materials and Methods

### Cell Culture

Adult rat spinal cord oligodendrocyte precursor cells (OPCs) were isolated from Fisher rats by immunopanning with an anti-O4 antibody (mouse IgM) as described previously (Cheng et al., 2007). Rat cortical OPCs were isolated from newborn Sprague Dawley rats with the anti-O4 mouse IgM antibody and rat anti-mouse IgM magnetic beads following a previously published procedure (Bankston et al., 2019). Cells were cultured on poly-l-lysine/laminin-coated plastic dishes (10,000 cells/cm^2^) in DMEM-F12 medium that was supplemented with 2.5 g/l NaHCO3, 1% N2 supplement, 2% B27 supplement, 1% penicillin/streptomycin, 0.01% BSA, 5 µg/ml insulin, 40 ng/ml FGF2 (Millipore), and 20 ng/ml PDGFaa. OL differentiation was induced by culturing OPCs in mitogen-free media supplemented with 40 ng/ml triiodothyronine (T3) for 72 h. Only low passage OPCs were used (passage number <8 or <15 for adult rat spinal cord or neonatal rat cortical OPCs, respectively). Such passage numbers did not compromise OL differentiation response confirming the maintenance of OPC identity (Supplementary Fig. S1) (Bankston et al., 2019; Kilanczyk et al., 2016; Kuypers et al., 2016).

### ER Stress Induction

TM or TG (Sigma-Aldrich) were dissolved in DMSO and added to culture media as indicated. DMSO was used as a vehicle control. In culture media, the final DMSO concentration never exceeded 0.2%.

### Cell Death Analysis

Cell death was determined using CytoTox-Glo^™^ Cytotoxicity Assay (cat# G9291, Promega Corporation, Madison, WI) following the manufacturer’s recommendations.

### mRNA Expression Analysis by qPCR

RNA was extracted using Trizol and cDNA was synthesized following standard methodology. Taqman DNA polymerase and ViiA 7 Real-Time PCR System (Applied Biosystems, Grand Island, NY) were used together with the following Taqman primers (*Fa2h*, Rn01426720_m1); *Olig2*, Rn06344794_s1; *Myrf*, Rn01454573_m1; *Zeb2*, Rn00497196_m1; *Olig1*, Rn00572904_s1; *Zfp488*, Rn06385176_m1, *Ugt8*, Rn01521831_m1; *Gapdh,* Rn01775763-g1, Thermo Fisher Scientific, Walthanm, MA). The ΔΔCT method was used for quantification; the normalizing transcript was *Gapdh*.

### Immunoblotting

Total protein was extracted from cell cultures using the SDS PAGE loading buffer (0.1 ml/35 mm plate). After SDS PAGE separation of the denatured total protein lysate (10 µl/sample) immunoblotting followed the previously described methodology (Ohri et al., 2011). The primary antibodies included anti-OLIG2 (Millipore, catalog # AB9610, RRID:AB_570666, 1:5000, Burlington, MA) and anti-β-tubulin (loading control, Sigma, catalog # T8328, 1:3000, St. Louis, MO). ECL and BioRad ChemiDoc MP Imaging System were used for signal visualization. Immunoblot quantifications were performed using NIH ImageJ software and TIFF-formatted image files.

### RNA Sequencing

RNA quality control, rRNA/mtRNA removal and library construction followed standard methodology. Sequencing was performed on the Illumina NextSeq500 with single reads of 76 bp utilizing the 500 High-output v2 (75 cycle) sequencing kit.

### Analysis of Transcriptome Data

Quality-filtered sequencing reads were aligned to the Rattus norvegicus reference genome assembly (rn6) using Tophat (version 2.0.4). Differentially expressed genes (DEGs) were identified for pairwise comparisons using Cuffdiff (version 2.1.1) and a false discovery rate-corrected p value (q) < 0.05. Both datasets were deposited at NCBI (accession numbers: GSE166220/TG/and GSE81792/TM/). Dataset overlap analysis was performed using the web-based Venn diagram tool (http://bioinformatics.psb.ugent.be/webtools/Venn/) to identify the overlap, followed by the exact hypergeometric probability significance test of the overlap using a web-based program (http://nemates.org/MA/progs/overlap_stats.html). For the overlap significance analysis, the average number of genes that were expressed in the analyzed datasets was used as the total transcriptome input. That number was defined as an average total number of identified transcripts that were expressed at least at the average levels of the least expressed but significantly affected transcripts in pairwise comparisons for the 8 h timepoint, when the greatest number of DEGs was detected (15,943 average for TM data, 15,775 average for TG data, 15,859 average for both data sets). Gene ontology analysis was performed using gProfiler (Raudvere et al., 2019). For analyzing the effects of ER stress on OPC/OL linage-specific transcriptome, the top 500 OPC or OL-linage-enriched transcripts that were identified in mouse brains at postnatal days 7 were downloaded from the brain RNASeq database (www.brainrnaseq.org) (Zhang et al., 2014). For OPC or OL linage-enriched transcripts, the background was set as all other brain cells except newly myelinating OLs or all other brain cells, respectively; minimum expression levels were set at FMPK values >10 (OPC) or >5 (OL-linage). Their rat orthologs that were identified with gProfiler (Raudvere et al., 2019) were used for overlap identification.

### Meta-Analysis of Previously Published Datasets

The OL translatome dataset generation has been recently described (Forston et al., 2023; Gao et al., 2023) (Accession number: GSE225308 and http://scigenedatabase.com/). Briefly, OL ribosome-associated mRNAs and total spinal cord mRNAs (all spinal cord cell samples) were isolated from the SCI epicenter region (moderate T9 contusive SCI, IH 50 kdyn, female OL RiboTag mice, 8–10 week old). Differential gene expression (SCI OLs vs. naïve mice OLs or OLs vs. all cell for each experimental group) was determined using DESeq2 with a false discovery rate-corrected p-value (q)<0.05. Transcripts that were significantly downregulated in the single cell RNASeq (scRNASeq)-determined DA2 transcriptomic profile within the main molecular subtype of mouse OLs (MOL5/6, 50%–70% of all OLs in the mouse hippocampus and corpus callosum) were extracted from the differentially expressed gene list (supplementary data in Pandey et al. (2022)). GO enrichment analysis of that list was performed using gProfiler (Raudvere et al., 2019).

### Statistical Analysis of Cell Death, qPCR and Immunoblotting Data

The ratiometric data including Cytoglo-assay-measured cell death, qPCR-determined mRNA levels and immunoblot-determined OLIG2 expression levels were analyzed using the two-tailed non-parametric Mann–Whitney *U*-test.

## Results

### ER Stressors Reduce OL Linage-Specific Gene Expression in OPCs

**Figure 1.**
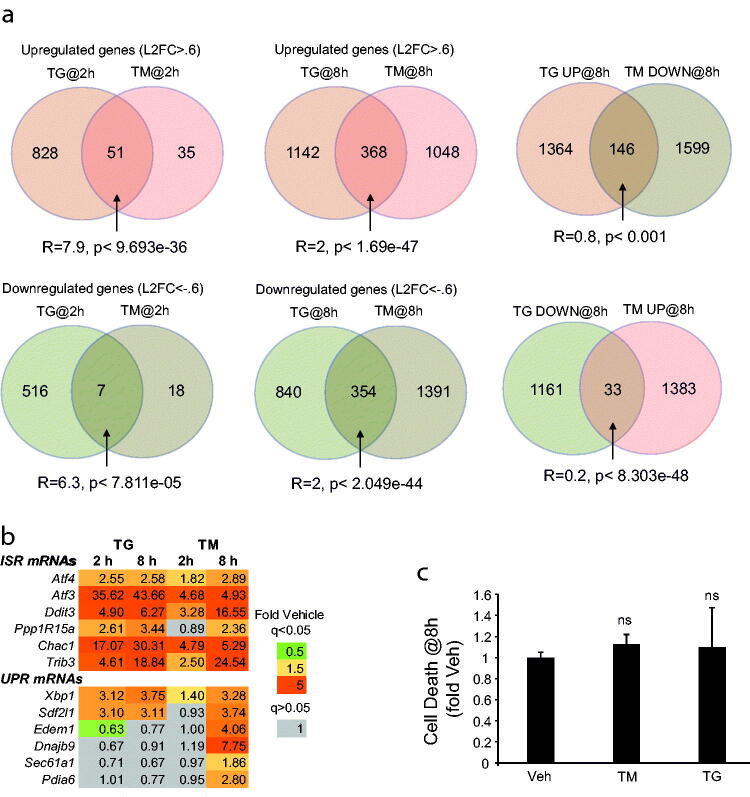
Widespread transcriptomic responses of OPCs to ER stress inducing drugs tunicamycin and thapsigargin before the terminal cytotoxicity of ER stress. Adult rat spinal cord OPCs were treated with 0.2 µM thapsigargin (TG), 5 µM tunicamycin (TM) or vehicle control (0.2% DMSO, veh) as indicated. (a,b), total RNA was isolated from TG, TM, or Veh-treated cells and analyzed by RNASeq. Identical samples from three independent experiments were used for this analysis. Differentially expressed genes (DEGs, q < 0.05) were identified based on comparisons to time matched veh controls (DEGs are listed in Supplementary Tables S1 and S2). (a), ven diagrams presenting numbers of commonly regulated or counter-regulated genes in the TG and TM data sets. Higher than expected overlaps are found for commonly upregulated and commonly downregulated genes (ratio between the number of identified overlapping genes and the expected number of randomly overlapping genes, R > 1). Lower than expected overlaps (R < 1) are observed for counter-regulated genes. *p* values of hypergeometric probability significance test are indicated. (b), expression heat map of ERSR marker transcripts. Similar induction of ERSR-ISR mRNAs by TG or TM. Maximal ERSR-UPR response followed 8 h TM treatment. (c) Vehicle-normalized changes in cell death were determined using the Cytotox-Glo assay. Note that after 8 h treatment, neither 0.2 µM TG nor 5 µM TM killed OPCs. Therefore, the OPC transcriptome data represent the ERSR stages that precede terminal toxicity. Data are mean ± SD of 6 sister cultures from 3 independent experiments; ns, *p* > .05, *u*-test.

The OPC transcriptome was analyzed by RNASeq after 2 or 8 h treatment with TG or TM. At 2 h, 879 or 86 mRNAs showed increases greater than log_2_ fold change (L2FC) 0.6 in TG or TM treated cells, respectively (Figure 1a; Supplementary Tables S1 and S2). Downregulations of L2FC<-0.6 were observed for 523 or 25 of TG- or TM-affected mRNAs (Figure 1a; Supplementary Tables S1 and S2). At 8 h, 1510 or 1416 TG or TM upregulated mRNAs and 1194 or 1745 TG or TM downregulated mRNAs were identified, respectively (Figure 1a; Supplementary Tables S1 and S2). There were significant overlaps between upregulated or downregulated transcripts by each ER stressor (Figure 1a). In addition, a lower than expected number of mRNAs showing opposite regulation with each drug is consistent with a similar transcriptomic response to either ER stress inducer (Figure 1a). However, while highly significant, the overlaps were only partial, with many transcripts upregulated or downregulated by only one drug (Figure 1a).

Similarities and differences were also evident when effects on the expression of 12 individual ERSR marker transcripts were analyzed. Specifically, both drugs strongly upregulated six ISR-induced mRNAs (Figure 1b). In contrast, 2 or 6 upregulated UPR-associated mRNAs were found in TG or TM treated OPCs, respectively (8 h treatments, Figure 1b). Importantly, after an 8 h TG or TM treatment, no significant increases of OPC cell death were found (Figure 1c). Hence, the transcriptome changes identified here precede the terminal cell death phase of the cytotoxic ERSR.

**Figure 2.**
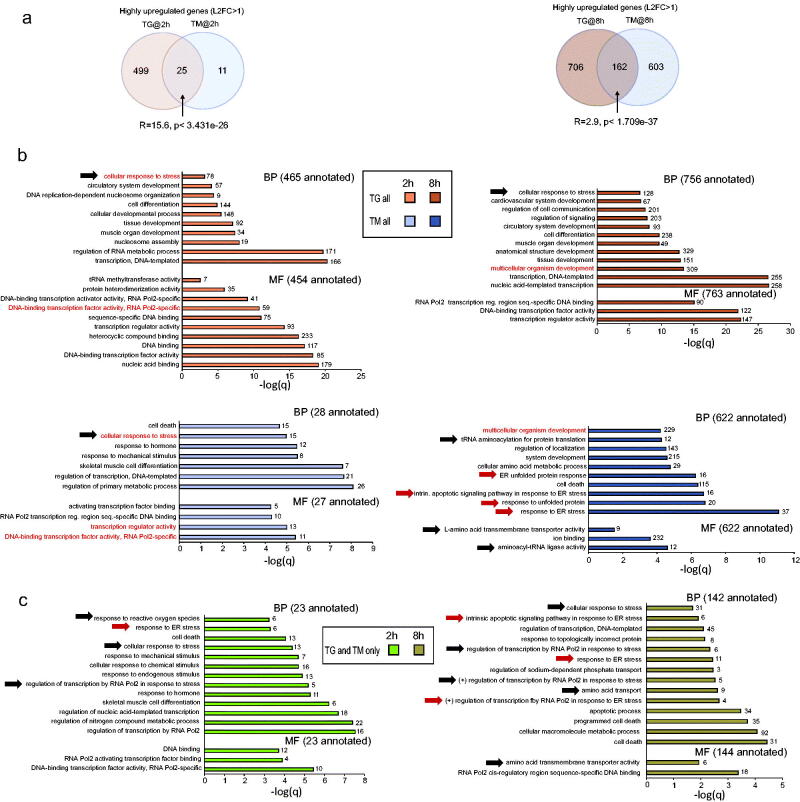
Gene ontology term (GO) enrichment analysis of highly upregulated genes indicates that tunicamycin is a more selective inducer of ER stress than thapsigargin. (a) Significant overlaps between highly upregulated genes in TG- and TM-treated OPCs (L2FC > 1). The representation factor (R) and *p* value of hypergeometric probability significance test are indicated. (b,c) Top enriched GOs (-log/q/is a ranking parameter of the enrichment; for clarity, some redundant and/or broadly defined terms are not shown; GO categories are indicated: BP- Biological Process, MF- Molecular Function including the number of annotated genes that were highly upregulated). Black or red arrows point GO terms associated with the general stress response/ISR or ER stress response/UPR, respectively. Numbers of genes from each GO that are upregulated are indicated at the bars. For each timepoint, enriched GOs that are shared between TM and TG-upregulated genes are marked in red. (b) Note the top enrichment for ER stress/UPR/ISR-associated GOs among TM upregulated transcripts after 8 h treatment. Only general stress/ISR GOs are enriched in the TG data set. Expanding GO analysis to include TG DEGs with L2FC > 0.6 did not affect those trends (Supplementary Table S3). (c) Commonly upregulated genes are enriched for ER stress and ISR GOs.

To evaluate the functional consequences of ER stress effects on the OPC transcriptome, we focused on highly regulated transcripts (L2FC > 1 or <-1). Overlaps of highly upregulated transcripts were strong at each time point (the relative overlap overrepresentation factor R = 15.6 or 2.9 after 2 or 8 h, respectively, Figure 2a). Gene ontology term (GO) enrichment analysis of the highly upregulated mRNAs in TG-treated OPCs revealed the greatest overrepresentation of GOs associated with mRNA transcription regulation regardless of treatment duration (Figure 2b). At both times, GOs related to cell differentiation/development and the general stress response, but not the ERSR, were also overrepresented (Figure 2b). The lack of ERSR GO enrichment was still observed even when the upregulation threshold was lowered to L2FC > 0.6 (Supplementary Table S3).

After the 2 h TM treatment, GOs that were overrepresented among the highly upregulated transcripts included mRNA transcriptional regulation and the general stress response (Figure 2b). At 8 h, several top enriched GO:Biological Process (GO:BP) categories were related to the ERSR including the UPR (Figure 2b). Also, the top-enriched GO: Molecular Function terms (GO:MF) included such ERSR/ISR-upregulated categories as aminoacyl-tRNA synthetases or amino acid transporters (Figure 2b) (Han et al., 2013; Krokowski et al., 2013).

For highly upregulated mRNAs that were shared by TG- or TM-treated OPCs, top enriched GOs at 2 h included those related to transcriptional regulation, general stress, as well as ERSR (Figure 2c). At 8 h, commonly upregulated mRNAs showed top enrichment for GOs related to cell death/apoptosis followed by the transcriptional response to ER stress, amino acid transport and the transcriptional response to general stress (Figure 2c).

**Figure 3.**
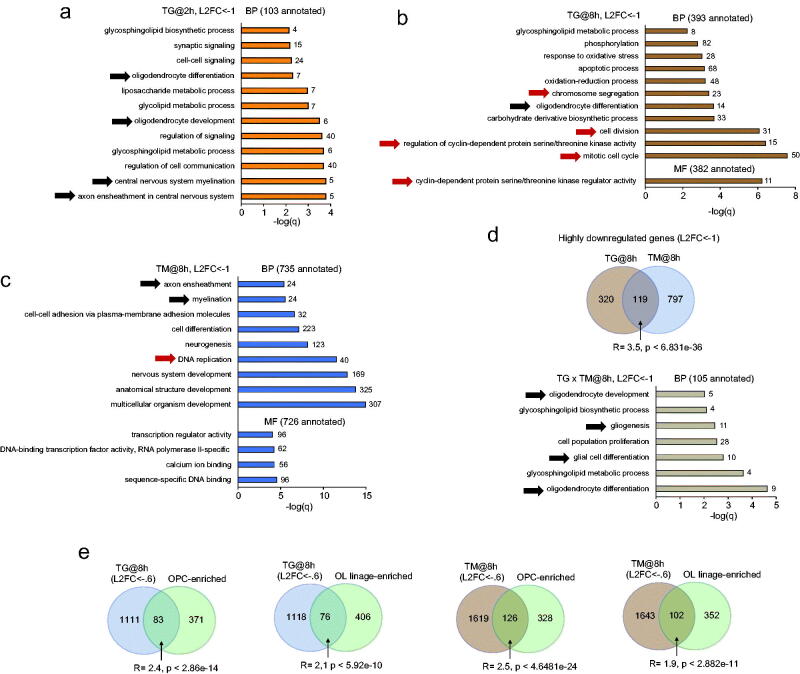
Reduced expression of genes associated with cell proliferation and OL linage-development in ER stressed OPCs. (a–d) GO enrichment analysis of highly downregulated genes (L2FC<-1). GO categories (BP or MF) and the number of downregulated genes with annotations are indicated. With low number of highly downregulated genes, no GO enrichment was identified for the 2 h TM treatment. Black or red arrows point to GOs related to OL linage development/differentiation/myelination or cell proliferation, respectively. (d) Transcripts that are commonly downregulated at 8 h show top enrichment for GOs related to OL linage differentiation/myelination and cell proliferation. (e) Overrepresentation of highly downregulated transcripts whose expression is enriched in OPCs or the entire OL linage (Zhang et al., 2014). Similar, significant overlaps were also observed for transcripts that were downregulated by the 2 h TG treatment (Supplementary Fig. S2). Conversely, no significant overlaps between TG/TM upregulated genes and OPC/OL lineage-enriched mRNAs were found (Supplementary Fig. S3). The representation factor (R) and *p* values of hypergeometric probability significance test are indicated.

In TG-treated OPCs, the top enriched GOs among highly downregulated mRNAs were related to cell cycle and glycolipid metabolism, as well as OL differentiation and myelination (Figure 3a,b). After 2 h TG treatment, the latter GOs showed the highest enrichment (Figure 3a). After 8 h treatment, TG-downregulated transcripts were most enriched for cell cycle-related GOs with continued overrepresentation of OL differentiation and glycolipid metabolism (Figure 3b). Few transcripts were downregulated after the 2 h TM treatment, resulting in no significant GO enrichment. Development-associated GOs topped the enrichment for downregulated mRNAs after 8 h TM exposure (Figure 3c). Those included CNS development, neurogenesis and myelination. At 8 h, the highly significant overlap between TM or TG downregulated mRNAs was enriched for OL differentiation, cell proliferation and glycolipid metabolism (Figure 3d). As glycolipids are major components of myelin, reduced expression of mRNAs associated with their metabolism is likely related to the negative effects of the ER stressor on OL differentiation/myelination.

Consistent with the negative effects of ER stress inducers on the expression of OL development-associated genes, there was a significant overlap between transcripts that are OPC or OL linage-enriched and those that are downregulated by the 8 h exposure to TG or TM (Figure 3d). For transcripts that were downregulated by the 2 h TG treatment, similar OPC/OL linage overlaps were identified (Supplementary Fig. S2). No significant overlaps were observed for the OPC or OL linage-enriched transcripts and TM/TG-upregulated transcripts at either 2 or 8 h (Supplementary Fig. S3).

Taken together, the ER stress inducers TM and TG trigger a partially overlapping transcriptomic response in OPCs. As expected, commonly upregulated mRNAs are enriched for GOs related to the general/ER stress responses and cell death. However, when the transcriptomic response to each drug is analyzed separately, ERSR GOs (including UPR) are significantly enriched for TM-, but not TG-, upregulated mRNAs. Finally, both TG and TM downregulate OL lineage-enriched mRNAs, many of which contribute to myelination. However, the number of downregulated OL linage genes is higher for TM than TG.

### Effects of ER Stress Inducers on Regulators of OL Identity

**Figure 4.**
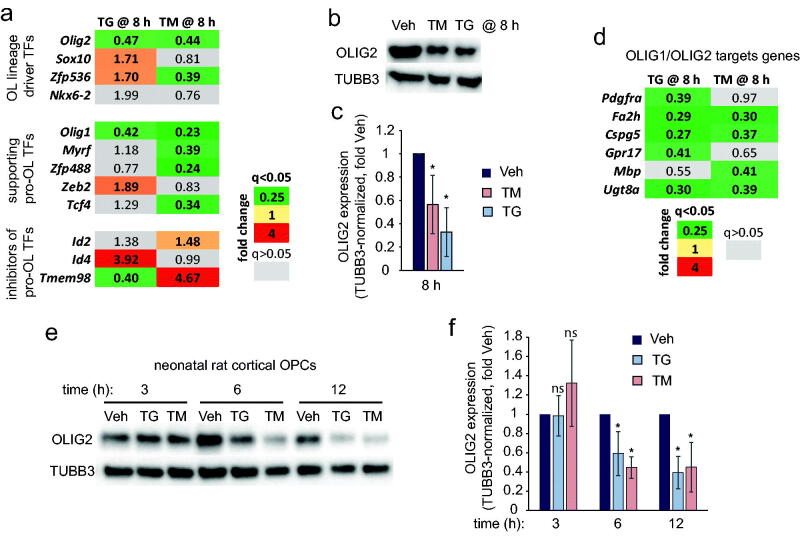
Reduced expression of the OL linage TFs in ER stressed OPCs. (a) effects of ER stress-inducing drugs on mRNAs for TFs that drive and/or regulate OL linage development. While *Olig1* and *Olig2* are downregulated by either drug, TM, but not TG, downregulated several pro-OL TF transcripts including *Zfp536*, *Myrf*, *Zfp488*, and *Tcf4*. In addition, transcripts for inhibitors of pro-OL TFs were upregulated (*Id2* and *Tmem98* by TM and *Id4* by TG). Expression data were retrieved from RNASeq results (Supplementary Tables S1 and S2). (b,c) Effects of ER stress inducing drugs on OLIG2 protein levels in adult rat spinal cord OPCs. Cells were treated with 0.2 µM TG or 5 µM TM or vehicle (veh, 0.1% DMSO) as indicated. Expression of OLIG2 protein was determined by Western blotting; β-tubulin (TUBB3) was used as a normalizer. A representative blot from one experiment is shown in (b); data in (c) represent blot quantifications from 3 independent experiments. (d) Examples of the common OLIG1/OLIG2 target effector genes that regulate OL linage development and are downregulated in RNASeq datasets from TG or TM-treated OPCs. (e,f) ER stress-associated downregulation of OLIG2 protein in neonatal rat cortical OPCs. Treatments included 0.5 µM TG, 2.4 µM TM or vehicle (veh, 0.1% DMSO) as indicated. A representative Western blot from one experiment is shown in (e); data in (f) represent blot quantifications from 3 independent experiments. Graphs in (c,f) depict averages ± SD (*p < .05; ns - *p* > .05; *u*-test).

The transcriptomic identity of the OL linage is established by several TFs including OLIG2, SOX10, ZFP536/ZFN536, and NKX6-2, with their combination being sufficient and each of them being necessary for OL linage induction (Najm et al., 2013; Yang et al., 2013). Hence, widespread downregulation of the OL linage-enriched transcripts in ER stressed OPCs may reflect reduced expression and/or activity of those TFs. Indeed, at 8 h, TG and TM reduced *Olig2* mRNA levels (Figure 4a). Moreover, in TG- or TM-treated OPCs, mRNAs for the OLIG2 inhibitors *Id4* or *Id2* (Samanta & Kessler, 2004) were upregulated, respectively (Figure 4a). *Id4* was also up after 2 h TG treatment (L2FC= 1.76606, q < 0.05). While the 8 h TM treatment reduced the expression of *Zfp536*, it was increased after the 8 h TG treatment (Figure 4a). TG, but not TM, also upregulated *Sox10* (Figure 4a). No significant effects of ER stressors were observed for *Nkx6-2*. Importantly, consistent with their negative effects on *Olig2* mRNA expression, the 8 h treatment of adult rat spinal cord OPCs with TM or TG lowered OLIG2 protein levels by 44% or 67%, respectively (Figure 4b,c). ER stress-induced downregulation of ZFP536 is also possible, although it may be reversed by Ca^2+^ signaling activation in TG-treated OPCs.

Additional TFs are required for CNS myelination acting at various stages of OPC differentiation/OL development. Those TFs, including OLIG1, MYRF, ZFP488, ZEB2, and TCF4, are regulated by and/or co-operate with the OL linage specification TFs such as OLIG2 or SOX10 (Emery & Lu, 2015; Wedel et al., 2020). Interestingly, the 8 h TM treatment reduced transcript levels of *Olig1*, *Myrf*, *Zfp488*, and *Tcf4* while upregulating *Tmem98* (Figure 4a), the latter of which encodes a negative regulator of MYRF (Huang et al., 2018). Of note, *Olig1*, *Myrf*, and *Zeb2* are all OLIG2 targets suggesting that lower OLIG2 expression/activity contributes to their downregulation (Yu et al., 2013). *Olig1* was also reduced by the 8 h TG treatment (Figure 4a). Conversely, *Zeb2* was up and *Tmem98* was down in TG-treated cells (Figure 4a). Consistent with ER stress-associated downregulation of *Olig1/Olig2* transcripts, TM or TG decreased mRNA levels of such common OLIG1/OLIG2 target genes as *Mbp*, *Utg8a*, *Fa2h*, and *Cspg5*, or *Pdgfra*, *Fa2h*, *Ugt8a*, *Cspg5*, and *Gpr17*, respectively (Figure 4d) (Weng et al., 2012). Thus, the TM-triggered ERSR includes the downregulation of several pro-myelination TFs and their targets. While in TG-treated OPCs, activated Ca^2+^ signaling may have interfered with the negative effects of ER stress on several pro-OL TFs, lower levels of OLIG2, *Olig1*, and at least some of their common targets were still observed. Finally, TG or TM downregulated OLIG2 in neonatal rat cortical OPCs with declines of 41–55% as early as 6 h after starting ER stress induction (Figure 4e,f). Therefore, ER stress is associated with reduced expression/inhibition of the critical OL linage TF OLIG2 that is observed in primary OPCs derived from various CNS regions at various stages of development.

### Reduced OL-Lineage-Specific Gene Expression in ER Stressed OLs

**Figure 5.**
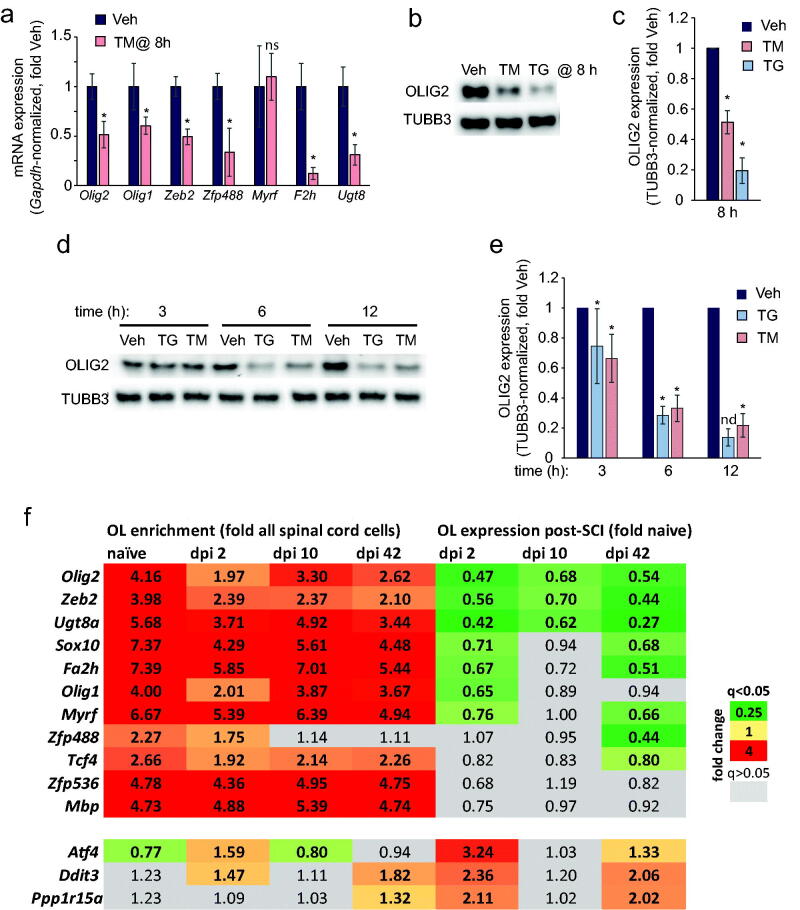
Reduced expression of the OL linage TFs in ER-stressed OLs. (a) Adult rat spinal cord OPC-derived OLs were treated with 5 µM TM or vehicle (veh). mRNA levels of OL TFs and their select targets were determined by qPCR with *Gapdh* used as a normalizer. (b,c) Adult rat spinal cord OPC-derived OLs were treated with 0.2 µM TG or 5 µM TM or vehicle (veh, 0.1% DMSO) as indicated. OLIG2 levels were determined by Western blotting with TUBB3 used as a loading control. (d,e) Reduced OLIG2 expression in neonatal rat cortical OPC-derived OLs that were treated with 0.5 µM TG or 2.4 µM TM or vehicle (veh, 0.1% DMSO) as indicated. A representative Western blot from one experiment is shown in (b,d); graphs in (c,e) represent blot quantifications. Data in (a,c,e) are averages ± SD from 3 independent experiments except the 12 h TG treatment in (e) where *n* = 2; *p < .05, *u*-test; nd, not determined. For (b,c), OPC differentiation efficiency was confirmed with 58.9 ± 1.9% O1^+^ OLs at the start of ER stress induction (Supplementary Fig S1). For (d,e), at least 40% O1 positivity rate was expected based on previous reports (Bankston et al., 2019; Kuypers et al., 2016). (f) The RiboTag-RNASeq analysis of mature OL translatome and RNASeq analysis of total spinal cord RNA (all cells) were performed using contused mouse spinal cord tissue at the indicated times after moderate T9 SCI (50 kdyn, IH) (Forston et al., 2023; Gao et al., 2023). Transcript expression data for OL TFs, their select targets, and ERSR markers are shown in the heat map. OL enrichment is defined as a ratio of mRNA expression between OL translatome and total RNA from all spinal cord cells in each set of paired samples from control- or SCI mice; OL expression is defined as a ratio of mRNA expression between OL translatomes from SCI mice vs. controls. Note OL-enriched expression of all OL lineage/OL identity genes (all cell-normalized OL translatome expression). Several of those OL-enriched mRNAs including *Olig2* show post-SCI downregulation that coincides with upregulation of ERSR/ISR marker mRNAs (*Atf4*, *Ddit3*, and *Ppp1r15a*). Data represent 3 biological replicates, each pooled from 2 animals (see materials and methods for additional information about the dataset).

The ER stress mediated downregulation of OL-lineage-specific gene expression was also observed in immature OL cultures that were derived from adult rat spinal cord OPCs. An 8 h treatment with TM reduced several OL-lineage enriched transcripts including *Olig1*, *Olig2*, *Zeb2*, *Zfp488*, *F2h*, and *Ugt8* (Figure 5a). Moreover, decreasing OLIG2 expression followed 8 h exposures to TM or TG with declines of 49% or 81%, respectively (Figure 5b,c). A similar response was observed in immature OLs that were derived from neonatal rat cortical OPCs (Figure 5d,e). Those findings indicate that ER stress inhibits OL-specific gene expression.

Acute/subacute activation of OL ERSR is well established after moderate contusive SCI (Ohri et al., 2011; Penas et al., 2007). Recent reports confirmed acute activation of the ISR arm of the OL ERSR/ISR by analyzing the OL-specific translatome changes after mouse T9 SCI on day post-injury (dpi) 2 (Forston et al., 2023; Gao et al., 2023). In addition, subchronic re-activation of the OL ERSR/ISR was reported on dpi 42 (Gao et al., 2023). Therefore, we re-analyzed the published OL translatome data to test whether the downregulation of OL-specific genes coincides with ERSR/ISR activation after SCI (Forston et al., 2023). Eight out of 11 ER stress-downregulated mRNAs for OL TFs or their OL-specific targets showed significantly reduced expression (Figure 5f). On both dpi 2 and 42, downregulation of the OL identity TFs *Olig2*, *Zeb2*, *Sox10*, and *Myrf* was accompanied by increased expression of ISR/ERSR genes (*Atf4*, *Ddit3/Chop* and *Ppp1r15a/Gadd34*, Figure 5f). In addition, reduced expression of *Olig1*, *Zfp488*, and *Tcf4* was found at one of those timepoints (Figure 5f). Of note, in both naïve and all SCI mice, OL-enriched expression of all those TFs was observed except of Zfp488, whose OL enrichment was statistically insignificant on dpi 10 and 42 (Figure 5f). Therefore, after SCI, ERSR/ISR activation correlates with reduced expression of genes that determine OL identity.

Recent single cell RNASeq (scRNASeq) analysis of the corpus callosum tissue from mouse models of Alzheimer’s disease or demyelination revealed several disease-associated, transcriptomic clusters of OLs (Pandey et al., 2022). The disease-associated cluster 2 (DA2) of the dominant mouse OL subtype MOL5/6 showed strong ERSR/ISR activation and was proposed to represent OLs under stress (Pandey et al., 2022). Meta-analysis of 499 downregulated genes in the DA2 cluster (L2FC MOL5/6 DA2 vs. MOL5/6< −0.6, q < 0.05) revealed significant enrichment for GO terms related to myelination (Supplementary Table S3). For instance, 32 genes of the GO:CC term myelin sheath (enrichment *p* = 5.21 × 10^−18^) showed a median downregulation by 40% (Supplementary Table S3). That group included the OL-specific mRNAs *Plp1*, *Cldn11*, *Cnp*, and *Mag*. Hence, in various white matter pathologies ranging from acute injuries to slowly progressing neurodegeneration ERSR/ISR activation is associated with decreases in OL-specific gene expression.

## Discussion

The presented RNAseq datasets reveal hundreds of transcripts that are differentially expressed in rat OPCs following treatment with ER stress inducers TG or TM. The scale of such a response is consistent with previous reports of extensive gene expression changes in various cells types that were exposed to those ER stress-inducing toxins (Han et al., 2013; Liu et al., 2016; Rendleman et al., 2018; Reich et al., 2020; Yang et al., 2021). However, GO analysis of the current datasets suggests that at least in OPCs, TM is a more selective inducer of ERSR than TG. ERSR-associated downregulation of the OL lineage-specific gene expression is another major finding from the current analysis. These inhibitory effects of ERSR are likely mediated by lower expression and/or activity of several major OL lineage TFs including OLIG2. Analysis of the mature OL translatome from the contused spinal cord revealed a similar downregulation of OL-lineage TFs that coincided with SCI-associated activation of ERSR/ISR. Finally, single cell OL transcriptomes from mouse models of Alzheimer’s disease or demyelination showed a subset of cells with highly activated ERSR and downregulation of OL-specific transcripts for various myelin components.

The higher selectivity of TM vs. TG to induce OPC ER stress may be attributed to distinct mechanisms of action of these two toxins. Specifically, as TG depletes ER Ca^2+^, it transiently increases cytosolic [Ca^2+^]_i_ and may activate cytosolic influx of Ca^2+^ via store operated Ca^2+^ channels (Foufelle & Fromenty, 2016; Pick et al., 2023; Razani-Boroujerdi et al., 1994). Therefore, TG may activate Ca^2+^ signaling, including Ca^2+^-regulated gene expression (Rodland et al., 1997). Such a Ca^2+^ driven gene expression program will likely dilute the ERSR-driven transcriptomic response to TG. Conversely TM appears to be a more selective ER stress inducer suggesting that at least in OPCs, the TM-mediated inhibition of N-glycosylation has relatively less non-ER stress consequences than the TG-mediated inhibition of the ER Ca^2+^ pump SERCA.

Interestingly, comparative analysis of TM vs. TG-treated human LN-308 astrocytoma cell line revealed only limited overlaps of their respective transcriptomes, translatomes and proteomes (Reich et al., 2020). After 2- or 6 h toxin exposure, transcriptomic overlaps of 19% or 43% were reported among upregulated genes, respectively (Reich et al., 2020). In OPCs, the overlaps for the upregulated transcripts at L2FC > 0.6 (>1.51 fold change) appeared to be even lower (5.5% at 2 h and 14% at 8 h, Figure 1). Importantly, in LN308 cells, like in OPCs, the commonly upregulated genes were highly enriched for ERSR/ISR/UPR-related GOs (Reich et al., 2020). While the extent of overlap between cellular responses to TM and TG is likely cell type-dependent, these comparative analyses indicate that not all effects of those commonly used ER stress inducers are ERSR/ISR/UPR-mediated. Such conclusions likely extend to other reagents that initiate ER stress by disrupting various aspects of ER homeostasis (Foufelle & Fromenty, 2016). Therefore, using more than one ER stress inducer would facilitate higher confidence identification of mediators and effectors of the ERSR/ISR/UPR. In OPCs, the relatively higher ER stress selectivity of TM vs. TG makes the former drug a reasonable choice for initial studies of the ERSR effects in OL lineage cells.

Various toxins including TM and TG are widely used as a convenient way to model disease associated ER stress in cultured cells or animals. While our transcriptomic data confirm ER stress induction in OL lineage cells that were treated with these drugs, limitations of such an ER stress modeling approach are also evident. Additional signaling modules are activated and secondary response to cellular damage other than ER impairment is also likely. Likewise, disease-associated ER stress such as that after SCI may be accompanied by yet another set of cell/tissue responses including hypoxia/ischemia, oxidative stress, or mitochondrial dysfunction (Whittemore et al., 2022). In each case, a different context of the ERSR may modulate its mediators and change its outcome. Such limitations are well exemplified by imperfect correlations between the effects of various genetic and/or pharmacological interventions that reduce TM/TG toxicity in OL linage cells vs. improved or unaffected white matter damage after SCI (Ohri et al., 2013; Ohri et al., 2014; Saraswat Ohri et al., 2023).

ERSR-mediated downregulation of OL lineage-enriched transcripts, including those for several OL-specific TFs, was an unexpected observation from the current analyses. One can speculate that chronic, excessive ERSR will likely reduce gene expression programs that are required for OPC differentiation into OLs and myelination by immature OLs (Emery & Lu, 2015). Such inhibition may impair myelin development and/or the OPC-driven myelin repair after white matter injury. In addition, as OL-specific TFs, including MYRF, are required for myelin sheath maintenance in adult animals, persistent inhibition of OL-specific gene expression may increase susceptibility to myelin degeneration (Koenning et al., 2012). While the consequences of a prolonged, ERSR-mediated inhibition of OL gene expression programs may be detrimental, a transient inhibition may support the survival of OLs under stress. As myelination is associated with high rates of lipid and protein biosynthesis, actively myelinating OLs are highly sensitive to ER stress (Lin & Popko, 2009). Consequently, decreasing pro-myelination gene expression could help restore ER homeostasis by reducing demand for biosynthetic processes that are ER-dependent.

Interestingly, ER stress-mediated inhibition of a cell type-specific gene expression program has been reported in pancreatic β-cells and hepatocytes. In both cases, ERSR-mediated inhibition of cell identity-determining TFs has been associated with partial de-differentiation. For instance, reduced insulin expression followed lower expression/activity of β-cell-specific TFs, including MAFA and PDX1 (Jang et al., 2011; Kim et al., 2011; Leenders et al., 2021). In hepatocytes, the entire network of hepatocyte-specific TFs was downregulated, including such hepatocyte identity drivers as HNF4A, NR1H4, or FOX2A (Dubois et al., 2020). In addition, induction of the transcriptional repressor NFIL3 participated in the hepatocyte de-differentiation response to ER stress (Dubois et al., 2020). Intriguingly, OL lineage cells, pancreatic β-cells and hepatocytes share high ER stress sensitivity due to intense usage of the ER and the secretory pathway (Ajoolabady et al., 2023; Lin & Popko, 2009; Sharma et al., 2021). Therefore, transient inhibition of various cell type-specific processes that put a heavy burden on the ER, may support the restoration of ER homeostasis and ensure survival of fully functional cells. However, under prolonged ERSR, persistent inhibition of those cell-specific functions could be detrimental. For instance, in pancreatic β-cells, a de-differentiation response to persistent ERSR has been proposed as a potentially pathogenic contributor to diabetes (Jang et al., 2011; Kim et al., 2011; Leenders et al., 2021).

Interestingly, complex interactions between pathological ER stress, Schwann cell differentiation and peripheral myelin degeneration were reported in a mouse model of Charcot–Marie–Tooth 1B disease due to toxic accumulation of the mutant protein P0. In that model, the upregulation of anti-differentiation TFs attenuated ERSR and improved the maintenance of myelin integrity (Florio et al., 2018). Conversely, Schwann cell-specific disruption of eIF2α phosphorylation enhanced the anti-differentiation response and worsened myelin pathology (Scapin et al., 2020). Hence, ER stress-associated chronic reduction of cell-type specific gene expression may be compensatory if such reduction does not go below a critical level of gene expression that is needed for tissue homeostasis.

Taken together, only partial transcriptome overlaps were identified in OPCs that were treated with two widely used ER stress inducers, TM and TG. While ERSR transcripts were upregulated by both agents, TM appears to be a more selective ERSR activator than TG. ERSR-associated inhibition of the OL lineage-specific gene expression has been found across various stages of OL development, including mature OLs from several mouse models of white matter pathology. Therefore, a persistent decrease of OL-specific gene expression due to pathological ER stress may affect the structural and/or functional integrity of the white matter in Alzheimer’s disease, multiple sclerosis and after traumatic SCI.

## Supplementary Material

Supplemental Material

Supplemental Material

Supplemental Material

## Abbreviations

DA: disease associated transcriptomic profile
DEG: differentially expressed genes
DPAGT1: dolichyl-phosphate N-acetylglucosaminephosphotransferase 1
EAE: experimental autoimmune encephalitis
eIF2α: eukaryotic initiation factor 2α
ER: endoplasmic reticulum
ERAD: ER-associated protein degradation pathway
ERSR: endoplasmic reticulum stress response
GO: gene ontology term
GO:BP: GO biological process term
GO:MF: GO molecular function term
IH: Infinite Horizon spinal cord impactor
ISR: integrated stress response
L2FC: log_2_ fold change
OL: oligodendrocyte
MS: multiple sclerosis
OPC: oligodendrocyte precursor cell
SERCA: sarcoplasmic/endoplasmic reticulum Ca^2+^-ATPase
SCI: spinal cord injury
scRNASeq: single cell RNASeq
TF: transcription factor
TG: thapsigargin
TM: tunicamycin
UPR: unfolded protein response.
